# Deceased donor kidney allocation: an economic evaluation of contemporary longevity matching practices

**DOI:** 10.1186/s12913-020-05736-y

**Published:** 2020-10-09

**Authors:** Sameera Senanayake, Nicholas Graves, Helen Healy, Keshwar Baboolal, Adrian Barnett, Matthew P. Sypek, Sanjeewa Kularatna

**Affiliations:** 1 Australian Center for Health Service Innovation, Queensland University of Technology, 60 Musk Ave, Kelvin Grove, QLD 4059 Australia; 2 Royal Brisbane Hospital for Women, Brisbane, Australia; 3 School of Medicine, University of Queensland, Brisbane, Australia; 4 Australia and New Zealand Dialysis and Transplant (ANZDATA) Registry, Adelaide, SA Australia

**Keywords:** Kidney allocation, Cost utility analysis, Longevity matching, QALY, Transplant

## Abstract

**Background:**

Matching survival of a donor kidney with that of the recipient (longevity matching), is used in some kidney allocation systems to maximize graft-life years. It is not part of the allocation algorithm for Australia. Given the growing evidence of survival benefit due to longevity matching based allocation algorithms, development of a similar kidney allocation system for Australia is currently underway. The aim of this research is to estimate the impact that changes to costs and health outcomes arising from ‘longevity matching’ on the Australian healthcare system.

**Methods:**

A decision analytic model to estimate cost-effectiveness was developed using a Markov process. Four plausible competing allocation options were compared to the current kidney allocation practice. Models were simulated in one-year cycles for a 20-year time horizon, with transitions through distinct health states relevant to the kidney recipient. Willingness to pay was considered as AUD 28000.

**Results:**

Base case analysis indicated that allocating the worst 20% of Kidney Donor Risk Index (KDRI) donor kidneys to the worst 20% of estimated post-transplant survival (EPTS) recipients (option 2) and allocating the oldest 25% of donor kidneys to the oldest 25% of recipients are both cost saving and more effective compared to the current Australian allocation practice.

Option 2, returned the lowest costs, greatest health benefits and largest gain to net monetary benefits (NMB). Allocating the best 20% of KDRI donor kidneys to the best 20% of EPTS recipients had the lowest expected incremental NMB.

**Conclusion:**

Of the four longevity-based kidney allocation practices considered, transplanting the lowest quality kidneys to the worst kidney recipients (option 2), was estimated to return the best value for money for the Australian health system.

## Background

Shortages of donor kidneys is a challenge faced by health systems globally [1]. The number of patients on waitlists is growing exponentially and in 2017 more than 100,000 patients were waiting for a kidney transplant in the United States [2]. This figure for the United Kingdom and Australia are around 4500 and 1000, respectively [3, 4]. However, the fact that the estimated number of transplants happened globally in 2017 is around 100,000, highlights the severe shortage of donor kidneys the world is experiencing [5].

Equity of access and maximising health benefits are often competing objectives in organ allocation systems [6]. Good donor kidney allocation decision making aims for equipoise between the two competing objectives. Healthcare planners have developed a range of different kidney allocation processes to balance these objectives.

The greatest graft life years of young donor organs is transplantation into younger recipients and is associated with cost savings [7]. Allocating elderly donor kidneys to younger recipients results in higher graft failure rates, increasing the flow of patients back to an already-burdened waiting list. Analysis of 9250 transplants that occurred over 12 years, indicated that by avoiding transplantation of young donor kidneys to elderly recipients, could have saved a total of 27,500 graft-years, with an estimated cost saving of 1.5 billion dollars [8]. Longevity matching is therefore a strategy implemented in some allocation systems. The idea is that kidneys from younger donors are prioritised for younger recipients and restricted for older groups. The goal in the system is matching the survival of a donor kidney with that of the recipient. Graft-life years are potentially maximised whilst recipients of all ages have the opportunity of transplantation [9]. Equal Opportunity Supplemented by Fair Innings (EOFI) is another allocation model proposed to balance equity and efficiency of kidney allocation [10].

Different ‘longevity matching’ strategies are in practice. According to the 2014 deceased donor kidney allocation policy in the United States, recipients in the best 20% of the estimated post-transplant survival (EPTS) received priority for offers of kidneys in the best 20% of the kidney donor profile index (KDPI) [11]. In Canada, kidneys of young donors are preferentially allocated to young recipients, minimizing young kidneys transplanted into elderly recipients [12]. The ‘Eurotransplant’ allocation program in Germany, the Netherlands, Austria, Belgium, Luxembourg, Slovenia, Croatia and Hungary stream elderly donor kidneys to elderly recipients [9]. The allocation system in the United Kingdom was revised in September 2019, incorporating donor-recipient risk indexes for allocation decisions [13].

At present, longevity matching is not part of the allocation algorithm for Australia. Under the current allocation policy, all kidneys are first considered in a national algorithm that prioritizes highly sensitized candidates, transplants with zero human leukocyte antigen (HLA) mismatches and addresses centre imbalances with well HLA matched transplants. If the organ is not transplanted on national allocation it proceeds to local allocation. There are five transplant regions within Australia and algorithms varying across jurisdictions but typically prioritise transplants with few HLA mismatches followed by allocation based on waiting time. If no suitable local candidate is found the organ is offered again nationally based on waiting time and sensitisation [14]. On average, 20% of the deceased donor kidneys are transplanted according to the national allocation system and the remaining 80% according to state allocation systems. A background review in 2014 indicated several concerns with the current allocation systems [15]. Extreme age mismatches between deceased donors and recipients leading to poor quality organs offered to young healthy candidates, and organ sharing and geographic disparities in waiting list access and waiting times were some among many issues highlighted. Given the growing evidence of survival benefit due to longevity matching based allocation algorithms, development of a similar kidney allocation system for Australia is currently underway [14].

The future allocation system has the potential to increase graft-life years and address the criterion of cost-effectiveness. This is important for Australia where the increasing burden of chronic kidney disease (CKD) is driving growth in CKD related healthcare costs [16].

The aim of this research is to estimate the change to costs and health outcomes arising from ‘longevity matching’ as compared to usual practice. In the Australian healthcare system The evidence generated will complement the development of a new kidney allocation system.

## Methods

A decision analytic model to estimate cost-effectiveness was developed using a Markov process. Four plausible competing allocation options were compared to the current kidney allocation option (Table 1). They are: allocating the best 20% of KDRI donor kidneys to the best 20% of EPTS recipients (option 1); allocating the worst 20% of KDRI donor kidneys to the worst 20% of EPTS recipients (option 2); allocating the youngest 25% of donor kidneys to the youngest 25% of recipients (option 3), which resembles Canadian allocation system; and, allocating the oldest 25% of donor kidneys to the oldest 25% of recipients (option 4) both of which resemble the Eurotransplant allocation system.

**Table 1:** Allocation methods compared in the study

Option	Allocation method
Current practice	Current kidney allocation method in Australia
1	Allocating the best 20% of KDRI donor kidneys (KDRI ≤0.9148) to the best 20% of EPTS (EPTS ≤1.033) recipients. Remaining 80% of the donor kidneys (KDRI > 0.9148) transplanted to remaining 80% of recipients (EPTS > 1.033) according the current allocation system
2	Allocating the worst 20% of KDRI donor kidneys (KDRI ≥1.7208) to the worst 20% of EPTS (EPTS ≥2.4806) recipients. Remaining 80% of the donor kidneys (KDRI < 1.7208) transplanted to remaining 80% of recipients (EPTS < 2.4806) according the current allocation system
3	Allocating the youngest 25% of donor kidneys (age of the donor ≤32 years) to the youngest 25% (age at transplant ≤41 years) of recipients. Remaining 75% of the donor kidneys (age of the donor > 32 years) transplanted to remaining 75% of recipients (age at transplant > 41 years) according the current allocation system.
4	Allocating the oldest 25% of donor kidneys (age of the donor ≥58 years) to the oldest 25% (age at transplant ≥60 years) of recipients. Remaining 75% of the donor kidneys (age of the donor < 58 years) transplanted to remaining 75% of recipients (age at transplant < 60 years) according the current allocation system

*KDRI* Kidney donor risk index, *EPTS* Estimated post-transplant survival

### Target population

The study population was Australian CKD patients who were waitlisted for a kidney transplant. Data from the Australia and New Zealand Dialysis and Transplant Registry (ANZDATA) [17] were used. ANZDATA collects and reports the incidence, prevalence and outcome of dialysis treatment and kidney transplantation for all patients with end stage kidney disease treated with kidney replacement therapy across Australia and New Zealand, including the private sector. Activities of the registry have been granted full ethics approval by the Royal Adelaide Hospital Human Research Ethics Committee (reference number: HREC/17/RAH/408 R20170927, approval date: 28/11/2017).

### Model structure

**Fig. 1:**
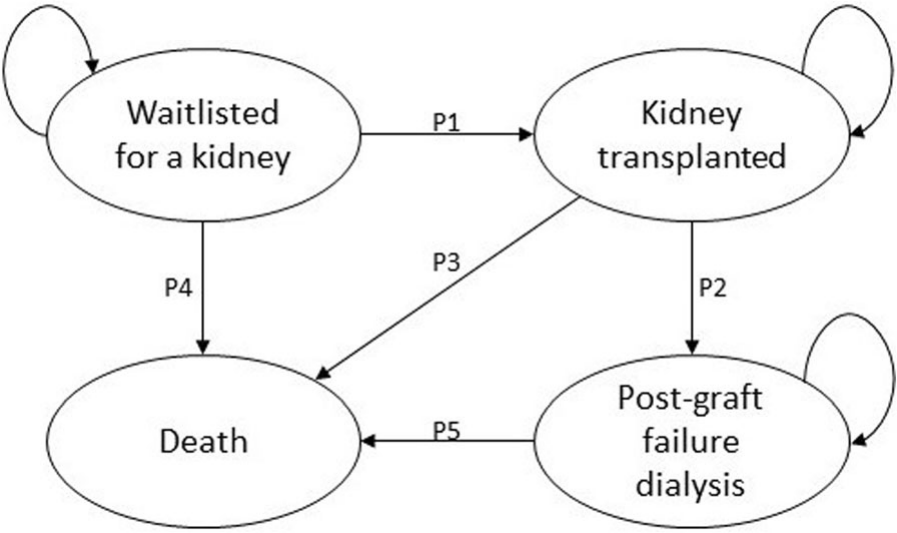
Markov model used. The Markov model has four health states: waitlisted for a kidney, kidney transplanted, post graft-failure dialysis and death. The cohort starts at the “waitlisted for a kidney” health state and patients in the cohort will be in this health state until they are transplanted or until die. When a patient transitions to the “kidney transplanted” health state they can experience either graft failure or death, or continuing successful transplantation

The Markov model (Fig. 1) was developed using TreeAge Pro 2019 (TreeAge Software, Inc., Williamstown, MA) to estimate the incremental costs and incremental quality-adjusted life years (QALY) associated with each donor organ allocation option. The model structure and assumptions used were validated by a systematic review [18] and by two clinical experts involved in kidney transplantation care in Australia.

For each option modelled patients may experience four health states: waitlisted, transplant, post graft-failure dialysis and death. The cohort starts at the “wait list” health state and patients in the cohort will be in this health state until they are transplanted or until die. The probability of being transplanted is different in each of the allocation options (Table 2). When a patient transitions to the “transplant” health state they can experience either graft failure or death, or continuing successful transplantation. It was assumed that once a graft fails patients will not be wait-listed for another transplant, thus, the patient will either remain on dialysis or die while on dialysis.

**Table 2:** Parameter estimates used in the model

Parameter	Current practice		Option 1		Option 2		Option 3		Option 4		Distribution	Level of evidence
	Baseline estimate	SEM	Baseline estimate	SEM	Baseline estimate	SEM	Baseline estimate	SEM	Baseline estimate	SEM		
**Transition probabilities**												
Probability of transplant while on waitlist												1
Lambda (λ)	0.2898	0.0184	0.2194	0.0225	0.3148	0.0222	0.3102	0.0292	0.3387	0.0243	Normal	
Gamma (ϒ)	1.544	0.0188	1.609	0.0237	1.481	0.0217	1.5982	0.0235	1.490	0.0227	Normal	
Age coefficient	−0.009	0.0011	−0.006	0.0017	−0.008	0.0013	−0.011	0.0016	−0.010	0.0014	Normal	
Graft failure												1
Lambda (λ)	0.1662	0.0199	0.2018	0.0359	0.1307	0.0187	0.1219	0.0232	0.1173	0.0179	Normal	
Gamma (ϒ)	0.4424	0.0158	0.4178	0.0180	0.4898	0.0212	0.4081	0.0185	0.4536	0.0204	Normal	
Age coefficient	0.020	0.0025	−0.021	0.0034	−0.019	0.0031	−0.013	0.0036	− 0.015	0.0033	Normal	
Mortality after transplant												1
Lambda (λ)	0.0020	0.0004	0.0023	0.0007	0.0022	0.0006	0.0021	0.0006	0.0018	0.0005	Normal	
Gamma (ϒ)	0.9348	0.0313	0.9410	0.0353	0.8720	0.0396	0.9407	0.0357	0.8841	0.0392	Normal	
Age coefficient	0.0475	0.0036	0.0465	0.0046	0.0457	0.0046	0.0479	0.0046	0.0521	0.0046	Normal	
Mortality while on waitlist^#^												
*Base case analysis*	0.0184		0.0184		0.0184		0.0184		0.0184			1
*Sensitivity analysis*	0.0184	0.0003	0.0184	0.0003	0.0184	0.0003	0.0184	0.0003	0.0184	0.0003	Beta	1
Mortality after graft failure^#^												
*Base case analysis*	0.1091		0.1091		0.1091		0.1091		0.1091			1
*Sensitivity analysis*	0.1091	0.0006	0.1091	0.0006	0.1091	0.0006	0.1091	0.0006	0.1091	0.0006	Beta	1
**Utility**												
Transplant	0.82; 95% CI (0.74–0.90)										Uniform	2
Dialysis	0.70; 95% CI (0.62–0.78)										Uniform	2
**Cost (in AUD)**												
Transplant (1st year)	99,968 (+/− 15%)										Uniform	1
Transplant (2nd year onwards)	13,916 (+/− 15%)										Uniform	1
Dialysis	81,689.34 (+/− 15%)										Uniform	1

λ – Lambda (rate parameter); ϒ – Gamma (shape parameter); *SEM* Standard Error of Mean, *CI* Confidence Interval

Lambda, Gamma and Age coefficients were calculated from Weibull regression method. Fixed transition probabilities^#^ were calculated from cumulative incident rates as described in the methods section

Option 1: Best 20% of KDRI donor kidneys transplanted to best 20% of EPTS recipients. Remaining 80% of the donor kidneys transplanted to remaining 80% of recipients according the current allocation system; Option 2: Worst 20% of KDRI donor kidneys transplanted to worst 20% of EPTS recipients. Remaining 80% of the donor kidneys transplanted to remaining 80% of recipients according the current allocation system. Option 3: Youngest 25% of donor kidneys transplanted to youngest 25% of recipients. Remaining 75% of the donor kidneys transplanted to remaining 75% of recipients according the current allocation system. Option 4: Oldest 25% of donor kidneys transplanted to oldest 25% of recipients. Remaining 75% of the donor kidneys transplanted to remaining 75% of recipients according the current allocation system

The measure of health benefits or the effectiveness of each allocation option are quality adjusted life years. The perspective of the analysis was that of the healthcare payer. Both future costs and QALY were discounted at an annual rate of 5%, as recommended by the Medical Services Advisory Committee’s Technical Guidelines, Australia [19].

### Data sources

The costs of a transplant were taken from the report “The economic impact of end-stage kidney disease in Australia - Projections to 2020” by Kidney Health Australia in 2010 [20]. The cost during the first year of deceased donor kidney transplantation was AUD $81,549. This included costs of the surgery, hospitalization, specialist consultations, immunosuppressive therapy and other drugs, as well as cost of the donor surgery. From year two onwards the annual cost for follow-up management which included immunosuppressive therapy, other drugs and non-drug follow-up costs was AUD $11,770 per year [20].

Dialysis costs were taken from the New South Wales Dialysis Costing Study (2008). The total dialysis cost included costs directly related to dialysis, such as nursing, allied health, dialysis fluid and consumables and depreciation costs, as well as ongoing CKD management costs, such as pharmacy, pathology and medical costs. Of the dialysis patients, 81% are on haemodialysis and the remainder are on peritoneal dialysis. Of the haemodialysis patients 68% are undergoing in-centre haemodialysis, 11% are on home haemodialysis and the remainder are undergoing haemodialysis at satellite centres [21]. The final dialysis cost of AUD $69,089 was calculated as a blend of all the dialysis modalities, proportionate to the different dialysis modalities practiced in Australia. All costs were converted to 2018 values.

Utility scores for different health states for Australian CKD patients are not available. Instead we took utility values for the transplant and dialysis states from a systematic review and meta-analysis [22]. The authors reviewed 190 studies reporting more than 300 utilities in more than 56,000 patients. The utility score for the transplant health state was estimated to be 0.82 (95% CI 0.74 to 0.90), while that of a dialysis health state was 0.70 (95% CI 0.62 to 0.78).

Two separate data sets were created to calculate the transition probabilities. The first dataset set was created by linking patients who were implanted with deceased donor kidneys (1st graft) between 1st January 2007 and 31st December 2017 in Australia to the waitlisted patient information dataset using a unique patient identification number provided by ANZDATA. The final dataset included those who were transplanted as well as those who were waitlisted but not transplanted during the study period. Furthermore, transplanted patients’ EPTS score and the KDPI of implanted organs were available. Both variables have been validated in Australian kidney transplant populations as valid measures of recipient and donor quality respectively [23, 24]. The probability of transplant and mortality while on waitlist and the probability of mortality and graft failure after transplantation was calculated in the first dataset population. The second dataset was created by linking CKD patients who started hemodialysis or peritoneal dialysis between 1st January 2007 and 31st December 2016, to transplant and waitlist patient information datasets. The probability of mortality while on dialysis after graft failure was calculated in the second dataset.

Transition probabilities of current practice were calculated from the first dataset. Then this was divided into overlapping subsets of data (option 1 to 4) for the purpose of the modelling. Note that data not conforming to the criteria of a particular hypothetical allocation option (Table 1) were excluded from a subset.

Three transition probabilities were calculated from each of the dataset populations:

- the annual probability of being transplanted from all waitlisted patients (Fig. 1; P1)
- the annual probability of graft failure following transplant (Fig. 1; P2)
- the annual probability of mortality following transplant (Fig. 1; P3)

Weibull regression was used to calculate above three probabilities based on its visual (parametric function fits well to the non-parametric function [25]) and statistical fit (based on Akaike and Bayesian information criterion (AIC, BIC) [26]) to the observed data. The Weibull distribution assumes that the baseline hazard is time-dependent, thus it allows the baseline hazard to increase or decrease over time at a different rate [27]. Age was considered a co-variate in the model. Lambda (λ), Gamma (ϒ) and beta coefficient of age were used to calculate the time-dependant probabilities (Table 1). Age was considered 50 for the analysis.

Fixed transition probabilities were calculated for mortality while waitlisted (Fig. 1; P4) and mortality while on dialysis after graft failure (Fig. 1; P5). These two probabilities were assumed to be the same for all five allocation options.

Fixed transition probabilities were calculated as follows; initially, cumulative incidence and standard error of the cumulative incidence were calculated for each of the transition probabilities. Then the cumulative incidences were first converted to rates and then to annual transition probabilities using following formulas [28].

$$ Cumulative\ incidence(p)\to \frac{\begin{array}{c}\mathbf{Rate}\left(\boldsymbol{r}\right)\\ {}-\ln \left(1-p\right)\end{array}}{T\ (Duraion)}\to {\displaystyle \begin{array}{c}\mathbf{Annual}\ \left(\boldsymbol{t}=\mathbf{1}\right)\ \mathbf{transition}\ \mathbf{probability}\\ {}1-{\mathit{\exp}}^{- rt}\end{array}} $$

The quality of the data used to inform model parameters was determined using the modified hierarchies of data sources for economic analyses [29]. The quality of data sources range from 1 to 6 with the highest quality of evidence ranked 1.

### Model evaluation

The outcomes of change to costs and change to QALY for each option were tested in a simulated cohort of 1000 patients for a 20-year time horizon. As the cohort transitions through different health states according to the calculated transition probabilities, both costs and utility are accumulated and aggregated at the end of 20 years to yield total costs and QALYs. The Incremental Cost Effectiveness Ratio (ICER) was calculated using:

$$ ICER=\frac{Cos{t}_{Hypothesised\ allocation\ method}-{ Cos t}_{Usual\ care}}{QAL{Y}_{Hypothesised\ allocation\ method}-{ QAL Y}_{Usual\ care}}=\frac{\varDelta\ Cost}{\varDelta\ Effectiveness} $$

An intervention is considered cost-effective if the ICER is less than the chosen Wiliness to pay (WTP) threshold. The WTP for a marginal QALY for Australia, based on life satisfaction as an indicator of utility to estimate the WTP value, is in a range between AUD 42,000 to AUD 67,000 [30]. A different threshold of AUD 28,000 was proposed recently [31] and this reflects the opportunity cost of additional healthcare expenditures under a constrained budget. For this analysis we used AUD28,000, and then conducted scenario analyses with thresholds of AUD 42,000 and AUD 67,000.

### Cost effectiveness analysis - sensitivity analyses

Probabilistic sensitivity analyse (PSA) was performed to capture the uncertainty of the parameters used in the model and their effects on cost-effectiveness [32]. PSA was performed using the Monte Carlo simulation method with 20,000 iterations. In PSA, the input parameters for costs, utilities and transition probabilities were defined as probability distributions to reflect the range of parameter uncertainty (Table 2). Since the cost data are not expected to be skewed, uniform distribution was used.

Net monetary benefit (NMB), which represents the difference between economic value of health benefits and the change in costs, was used to evaluate the sensitivity of the cost-effectiveness results. The equation is:

$$ NMB=\left( WTP\times QALY\right)- Costs $$

NMB can be calculated either as an absolute parameter for a single option (eg: intervention) without any comparison or as an incremental parameter as indicated below [33].

$$ Incremental\  NMB= Absolute\ {NMB}_{Hypothesised\ allocation\ method}- Absolute\ {NBM}_{Usual\ care} $$

The average incremental NMB across 20,000 iterations was calculated for each pair of options compared (each allocation method versus current practice) in the analysis. A positive incremental NMB indicates a cost-effective decision and choosing anything else would incur an opportunity cost. The allocation option with the highest incremental NMB is the most cost-effective allocation strategy, compared to current practice, for Australia and choosing anything else would incur an opportunity cost. The probability of error in this decision was computed. The proportion of iterations in which an allocation method returns the highest NMB, compared to the current method, represents the probability that the particular option is the optimal decision. One minus this proportion (probability of error) indicates the probability that the allocation method does not yield the highest NMB compared to the current practice, thus the decision is incorrect [34].

## Results

### Quality of kidney transplanted is different across the allocation options

**Fig. 2:**
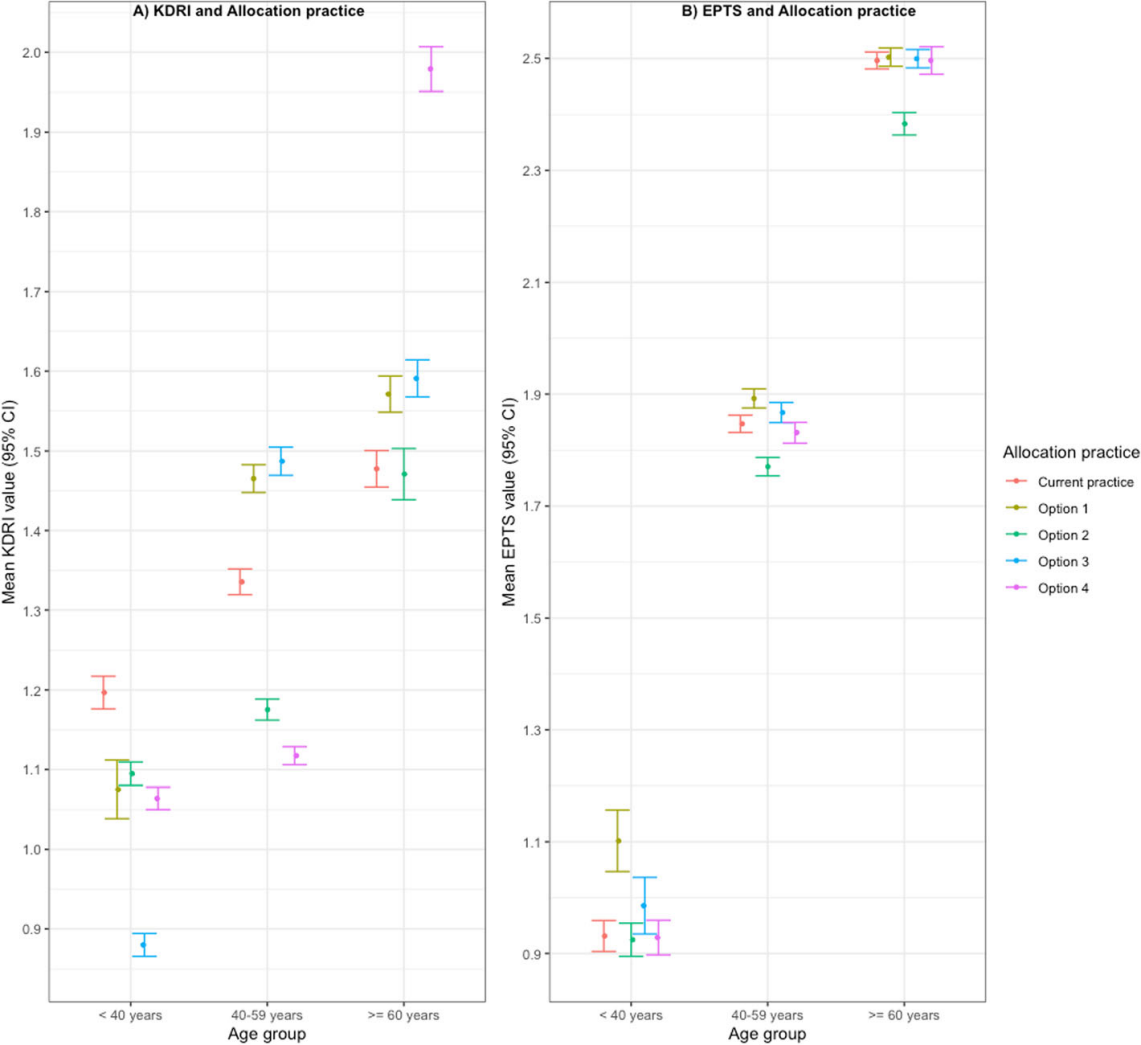
Mean KDRI and EPTS values according to different allocation practices. **a** Distribution of the mean KDRI values with 95% confidence interval according to different age groups and different allocation options. **b** Distribution of the mean EPTS values with 95% confidence interval according to different age groups and different allocation options

In comparison to current Australian practice, all the other allocation options had lower KDRI values among the younger (age at transplant ≤40 years) recipients (Fig. 2). However, among the older recipients (age at transplant ≥60 years) the KDRI value was higher in all the options except option 2 (allocating the worst 20% of KDRI donor kidneys to the worst 20% of EPTS recipients).

The mean EPTS score in allocation option 1 was significantly higher, compared to current Australian practice, among those who were less than 40 years at transplant (0.17; 95% CI 0.10 to 0.23) and those who were between 40 to 59 years at transplant (0.05; 95% CI 0.02 to 0.06). However, in option 2, compared to current allocation practice, the mean EPTS score was significantly lower among recipients who were between 40 to 59 years at transplant and those who were more than or equal to 60 years old at transplant.

### Cost-effectiveness results

Base case analysis indicates that options 2 and 4 are dominant (both cost saving and more effective) compared to the current allocation practice (Table 3).

**Table 3:** Summary statistics for age and kidney related indicators and cost-effectiveness results in the base-case analysis

Option	Cost-effectiveness results – base case analysis			
	Total cost (2018 AUD in Millions)^**a**^	Total effect^**a**^	Cost per QALY^**a**^	ICER (AUD per QALY)
Current practice	407.4	8471	48,096	
1	413.3	8399	49,205	- 81,944 (More costly, and less effective)
2	405.3	8616	47,034	Dominant (Cost saving and more effective)
3	405.3	8444	47,993	77,777 (Less costly and less effective)
4	405.9	8521	47,633	Dominant (Cost saving and more effective)

^a^Results presented for 1000 patients for 20-year time horizon

Dominant: Option is both cost saving and more effective

Cost per QALY = Cost / Effect

ICER = Incremental cost (Cost _Option 1,2, 3,4_ - Cost _current practice_) / Incremental effect (Effect _Option 1,2, 3,4_ - Effect _current practice_)

**Fig. 3:**
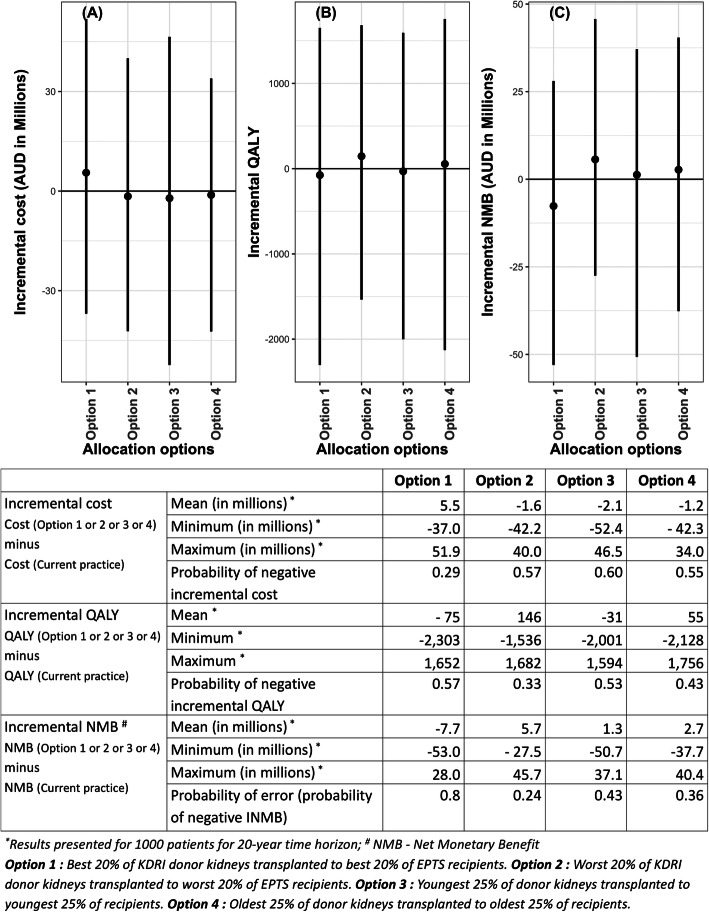
Mean and range of the incremental cost, QALY and NMB for each allocation options compared with current practice; **a** Incremental cost, **b** Incremental QALY, **c** Incremental NMB. The black vertical line in all three graphs indicate the range of values generated from the 20,000 iterations in PSA. Negative incremental cost (**a**) indicates a cost saving compared to current practice. The option with the highest probability of negative incremental cost has the most probability of being cost saving compared to current practice. Negative incremental QALY (**b**) indicates less effectiveness compared to the current practice and the option with the lowest probability of negative incremental QALY has the most probability of being effective compared to current practice. Negative incremental NMB (**c**) indicates the option is not cost effective compared to current practice. Probability of error indicated the probability of the option not being the cost-effective option compared to current practice. Therefore, the option with the lowest probability of error is the most suitable option. ^*^Results presented for 1000 patients for 20-year time horizon; ^#^ NMB - Net Monetary Benefit. Option 1: Best 20% of KDRI donor kidneys transplanted to best 20% of EPTS recipients. Option 2: Worst 20% of KDRI donor kidneys transplanted to worst 20% of EPTS recipients. Option 3: Youngest 25% of donor kidneys transplanted to youngest 25% of recipients. Option 4: Oldest 25% of donor kidneys transplanted to oldest 25% of recipients.

The impacts on cost, QALY and NMB outcomes of different allocation options compared to current practice, with all parameter uncertainties included, is shown in Fig. 3.

Compared to current practice, all the options, except option 1, were cost saving with a high degree of certainty. The highest QALY gain was from option 2 (QALY 146). Furthermore, option 2 returned the highest expected incremental NMB (5.7 million) and represented the optimal choice given current information. The uncertainty of this conclusion was estimated as 24%, although we note this does not include model uncertainty. Option 4 returned a positive incremental NMB (2.7 million), indicating that, compared to current practice, option 4 is most likely cost effective as well (probability of error 0.36). However, compared to option 2, option 4 was more costly, less effective and returned less net monetary benefits. Option 1 has the lowest expected incremental NMB (− 7.7 million), indicating that it’s the worst choice given current information. There is a considerable amount of uncertainty (43%) around the net monetary benefit of allocating the youngest 25% of donor kidneys to the youngest 25% of recipients (option 3).

Separate analysis was conducted against two different WTP thresholds, AUD 42,000/QALY and AUD 67,000/QALY to assess the sensitivity of the results. Neither of the WTP values changed the order of the average NMB of the different allocation options. Results when all the costs were converted to the United States dollars (2018), are presented in supplementary material.

## Discussion

This study is the first ever economic evaluation conducted to assess the cost-effectiveness of different contemporary longevity-based kidney donor allocation protocols. Our evaluation found that allocating the worst 20% of KDRI donor kidneys to the worst 20% of EPTS recipients returns the highest NMB. This option, along with allocating the oldest 25% of donor kidneys to the oldest 25% of recipients, is estimated to be more cost-effective compared with the current kidney allocation practices in Australia. Interestingly, transplanting the best 20% of KDRI donor kidneys to the best 20% of EPTS recipients, which resembles the United States allocation system, returned the lowest average NMB.

Under the current kidney allocation system in Australia a majority of the kidneys (80%) are allocated according to the state-base allocation system (compared to national allocation system). The primary driver of the state-base allocation system is the waiting time, which does not account for the reality that hazard ratios differ between patients on the waitlist [14]. The principle behind “longevity matching” is to allocate the grafts with the longest expected functioning graft-years to the recipients who are expected to live the longest [35]. The net benefit is overall reduced graft failure rates thus reducing patients returning back to the waiting list for a re-transplantation. Patients waiting for a re-transplant make-up about 10 to 15% of waitlist candidates [36]. Longevity matching has the potential to reduce this burden by reducing the number of re-transplants, in-turn expanding the population who receive a first transplant kidney [35].

Our study found that allocating the best kidneys to the best recipients was more costly and less effective compared with current Australian allocation practice. A recent Australian study came to the same conclusion [37]. This modelling study indicated that though allocating best kidneys to best recipients increases QALY in younger recipients, it does not increase QALY across all age groups. Our study found that though option 1 reduces the mean KDPI score among the recipient who is less than 40 years old, which are about 25% of all the transplants in Australia, the mean KDPI score was significantly increased among older ie > 40 years old recipients. This group contributes to around 75% of transplants in Australia [21]. Furthermore, the mean EPTS score was significantly higher in option 1 compared with the current practice, across all age categories. This, along with high mean KDRI in a majority of recipients in option 1, translated into increased graft failure and mortality probability among recipients in option 1 in our study.

Furthermore, it should be noted that in option 1, the best 20% of the recipients received the best 20% of donor kidneys and the rest of the 80% of recipients received the balance 80% of donor kidneys according to the current Australian kidney allocation system. Further, the option 1 was compared with the current allocation system in Australia, which is different to the previous kidney allocation system in United States. Therefore, the results of option 1 is not directly comparable with either the new or the previous allocation system in United States. A recent economic evaluation revealed that the new United States allocation policy is cost effective compared with the old policy with a marginal lifetime increment in QALY [38]. Furthermore, the new system is associated with higher total in-hospital costs and readmission rates [39], but better in addressing ethnic disparities in kidney allocation compared to the previous system [40]. In short, allocating best kidneys only to best recipients might not return the best value for money for Australia. This in-depth analysis may guide the policy development in Australia.

Our analysis estimates that transplanting the worst 20% of KDRI donor kidneys to the worst 20% of EPTS recipients and transplanting the oldest 25% of donor kidneys to the oldest 25% of recipients (old-to-old) are cost-effective allocation strategies compared with current Australian practice. The latter resembles the Eurotransplant Senior Program (ESP) implemented in 1997 with aims of improving the efficiency of the usage of old aged donor kidneys and reducing the waiting time for elderly patients [41]. Demand for dialysis is reduced as elderly recipients enjoy a significant improvement in life expectancy after transplantation [42]. A 5-year analysis of the ESP reported graft and patient survival had not been negatively affected compared with historical allocation practices. A consequence of ESP has been a doubling of elderly donor kidneys and reduction in waiting time of more than a year in elderly recipients [43].

Several other studies demonstrated the survival benefit of transplanting marginal quality kidneys into elderly recipients. Jay et al. (2017) demonstrated that transplanting a KDPI over 85% kidney into a recipient more than 60 years is associated with lower mortality hazard compared to waiting on the waitlist in hope of receiving a KDPI under 85% kidney [44]. According to Massie et al. (2014) the survival benefit of high-KDPI transplants was greatest in patients aged more than 50 years [45]. Furthermore, transplantation of marginal quality kidneys will reduce the kidney discard rates [46]. The literature is less informative about overall cost-effectiveness of these policies. This study directly addresses systems level cost effectiveness of different donor kidney allocation options in a context where Australia is currently in the process of developing a new kidney allocation policy.

Factors that improve the survival of a low-quality graft, could change the cost-effectiveness results of different allocation options discussed in the paper. Novel immunosuppressive drugs have the potential to improve graft survival significantly [47]. Furthermore, factors such as warm ischaemia time [48], recipient factors such as EPTS score, level of allosensitization and the cause of renal failure, and donor factors such as age, cause of death, donation after cardiac death, terminal creatinine have been found to be associated with the cost of kidney transplantation. Since these factors are also associated with graft-survival, they could influence the cost-effective results of the allocation options [49].

The current study has several limitations. Firstly, all the transition probabilities used in different allocation practices were sourced from a retrospective data set. This may not reflect the reality of current day allocation practices in Australia. Characteristics impacting graft and patient survival may have changed over the more than decade of assembly of the retrospective data set. Organ acceptance behaviour, availability of donor kidneys, KDRI profile of the donor pool and EPTS profile of the recipient pool are just some examples of change. However, modelling these and future changes after a radical change in the allocation practice is beyond the scope of this study. Thus, the authors believe the current study is done with the best available information at present. Secondly, the model considered recipients who had their first kidney transplantation and those who had re-transplantations were not accounted for. Thirdly, implementation of a longevity matching based kidney allocation system could increase the cost of transportation of organs, but this was not modelled in the analysis. Fourthly, we assumed that those who are waitlisted will either remain waitlisted, die or transplanted. However, in reality some waitlisted patients can be taken off the list temporarily or indefinitely, mostly due to other competing health events. This has not been modelled in this analysis.

## Conclusion

Longevity-based matching, including transplanting the worst 20% of KDRI donor kidneys to worst the 20% of EPTS recipients, was estimated to return the best value for money for the Australian health system. However, further research is warranted to assess how this option ensures equity of access to donor kidneys.

## Supplementary information

**Additional file 1.**

## Data Availability

The datasets used and/or analysed during the current study are available from the corresponding author on reasonable request.

## Abbreviations

ANZDATA: Australia and New Zealand Dialysis and Transplant Registry
AUD: Australian dollars
CKD: Chronic Kidney Disease
EPTS: Estimated Post-Transplant Survival
ESP: Eurotransplant Senior Program
HLA: Human Leucocyte Antigen
KDRI: Kidney Donor Risk Index
KDPI: Kidney Donor Profile Index
NMB: Net Monetary Benefits
PSA: Probabilistic Sensitivity Analyse
QALY: Quality-Adjusted Life Years
US: United States
WTP: Willingness to pay
