# Supplementary material for: Deceased donor kidney allocation: an economic evaluation of contemporary longevity matching practices
Source: BMC Health Serv Res. 2020 Oct 9;20:931. doi: 10.1186/s12913-020-05736-y (PMC7547436; doi:10.1186/s12913-020-05736-y)
Supplement: Supplementary file 1 — Additional file 1. [file 12913_2020_5736_MOESM1_ESM.docx]

**Supplementary material**

Results when all the costs were converted to the United States dollars (2018), are presented in supplementary table 1 and supplementary figure 1. Willingness to pay (WTP) threshold was US $ 19,471.

**Supplementary Table 1 :** Cost estimates used in the analysis

| **Description** | **Cost (US $)** |
| --- | --- |
| Transplant (1st year) | 69,519 |
| Transplant (2nd year onwards) | 9,677 |
| Dialysis | 56,807 |

**Supplementary Table 2 :** Summary statistics for age and kidney related indicators and cost-effectiveness results in the base-case analysis

| **Option** | **Cost-effectiveness results – base case analysis** | | | |
| --- | --- | --- | --- | --- |
|  | **Cost (2018 US $ in Millions)^*^** | **Effect^*^** | **Cost per QALY^*^** | **ICER (US $ per QALY)** |
|  |  |  |  |  |
| Current practice | 281.1 | 8,471 | 33,186 |  |
| 1 | 285.2 | 8,399 | 33,951 | - 55,906 (More costly, and less effective) |
| 2 | 279.6 | 8,616 | 32,453 | Dominant |
| 3 | 279.6 | 8,444 | 33,115 | 55,801 (Less costly and less effective) |
| 4 | 280.1 | 8,521 | 32,867 | Dominant |

*^*^Results presented for 1000 patients for 20-year time horizon;*

*Dominant : Option is both cost saving and more effective*

*ICER = Incremental cost (Cost _Option 1,2, 3,4_* - *Cost _current practice_) / Incremental effect (Effect _Option 1,2, 3,4_ - Effect _current practice_)*


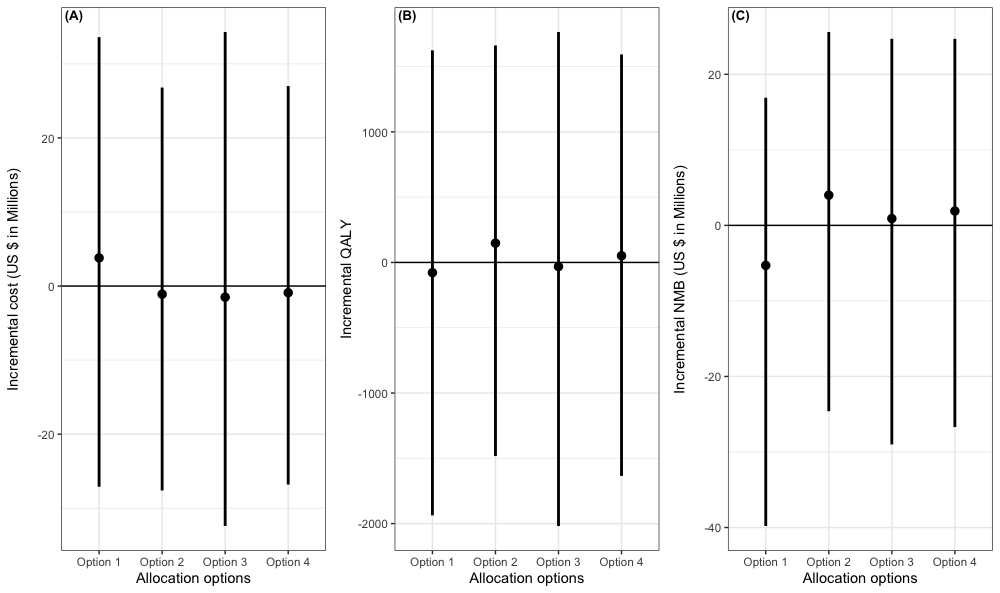


|  | | **Option 1** | **Option 2** | **Option 3** | **Option 4** |
| --- | --- | --- | --- | --- | --- |
| **Incremental cost**  **(Cost _Option 1 or 2 or 3 or 4_ minus Cost _Current practice_)** | Mean (in US$ millions) *^*^* | 3.8 | -1.1 | -1.5 | -0.9 |
|  | Minimum (in US$ millions) *^*^* | -27.1 | -27.6 | -32.4 | -26.8 |
|  | Maximum (in US$ millions) *^*^* | 33.6 | 26.8 | 34.3 | 27.0 |
|  | *Probability of negative incremental cost* | 0.29 | 0.57 | 0.60 | 0.55 |
| **Incremental QALY (QALY _Option 1 or 2 or 3 or 4_ minus QALY _Current practice_)** | Mean *^*^* | - 75 | 146 | -31 | 55 |
|  | Minimum *^*^* | -2,303 | -1,536 | -2,001 | -2,128 |
|  | Maximum *^*^* | 1,652 | 1,682 | 1,594 | 1,756 |
|  | *Probability of negative incremental QALY* | 0.57 | 0.33 | 0.53 | 0.43 |
| **Incremental NMB ^#^**  **(NMB _Option 1 or 2 or 3 or 4_ minus NMB _Current practice_)** | Mean (in US$ millions) *^*^* | -5.3 | 4.0 | 0.9 | 1.9 |
|  | Minimum (in US$ millions) *^*^* | -39.8 | -24.6 | -29.0 | -26.7 |
|  | Maximum (in US$ millions) *^*^* | 16.9 | 25.5 | 24.6 | 24.6 |
|  | *Probability of error (probability of negative INMB)* | 0.81 | 0.23 | 0.43 | 0.36 |

*^*^Results presented for 1000 patients for 20-year time horizon; ^#^ NMB - Net Monetary Benefit*

***Option 1 :*** *Best 20% of KDRI donor kidneys transplanted to best 20% of EPTS recipients.* ***Option 2 :*** *Worst 20% of KDRI donor kidneys transplanted to worst 20% of EPTS recipients.* ***Option 3 :*** *Youngest 25% of donor kidneys transplanted to youngest 25% of recipients.* ***Option 4 :*** *Oldest 25% of donor kidneys transplanted to oldest 25% of recipients.*

**Supplementary Figure 1 :** Mean and range of the incremental cost, QALY and NMB for each allocation options compared with current practice; A) Incremental cost, B) Incremental QALY, C) Incremental NMB

*The black vertical line in all three graphs indicate the range of values generated from the 20,000 iterations in PSA. Negative incremental cost (A) indicates a cost saving compared to current practice. The option with the highest probability of negative incremental cost has the most probability of being cost saving compared to current practice. Negative incremental QALY (B) indicates less effectiveness compared to the current practice and the option with the lowest probability of negative incremental QALY has the most probability of being effective compared to current practice. Negative incremental NMB (c) indicates the option is not cost effective compared to current practice. Probability of error indicated the probability of the option not being the cost-effective option compared to current practice. Therefore, the option with the lowest probability of error is the most suitable option.*
